# Toxicological analyses of the venoms of Nigerian vipers *Echis ocellatus* and *Bitis arietans*

**DOI:** 10.1186/s41182-024-00581-9

**Published:** 2024-01-29

**Authors:** Emeka John Dingwoke, Fatima Adis Adamude, Aliyu Salihu, Mujitaba Suleiman Abubakar, Abdullahi Balarabe Sallau

**Affiliations:** 1 Department of Tropical Diseases, UNESCO-International Center for Biotechnology, University of Nigeria, Nsukka, Enugu State Nigeria; 2 Department of Biochemistry, Faculty of Sciences, Federal University, Lafia, Nasarawa State Nigeria; 3 Department of Biochemistry, Faculty of Life Sciences, Ahmadu Bello University, Zaria, Kaduna State Nigeria; 4 Department of Pharmacognosy and Drug Development, Faculty of Pharmaceutical Sciences, Ahmadu Bello University, Zaria, Kaduna State Nigeria; 5 Venom, Antivenom and Natural Toxins Research Centre, Ahmadu Bello University, Zaria, Kaduna State Nigeria

**Keywords:** Snake venom, Snake venom metalloproteinases, Snake venom serine proteases, Snake venom phospholipase A_2_, Toxicological assay

## Abstract

**Background:**

Among the medically important snakes in Nigeria, *Echis ocellatus* and *Bitis arietans* have the most lethal venom. These venoms were classified according to the presence of snake venom metalloproteinases (SVMPs), snake venom phospholipase A_2_ (PLA_2_s), and snake venom serine proteases (SVSPs). Toxicological analyzes were performed to understand the significance of different protein families in venoms.

**Methods:**

Proteins were separated from venom using column chromatography. The skin and footpad of mice were used to determine hemorrhagic and edematogenic activities. Caprine blood plasma was used to test fibrinolytic activity in vitro.

**Results:**

The results showed that, compared to the crude venom, the SVMP fraction induced hemorrhagic effects with a diameter of 26.00 ± 1.00 mm in *E. ocellatus* and 21.33 ± 1.52 mm in *B. arietans*. Both SVSP and SVMP had anticoagulant effects; however, the SVSP fraction had a stronger effect, with a longer anticoagulation time of 30.00 ± 3.00 min in *E. ocellatus* and 26.00 ± 2.00 min in *B. arietans*. These main venom toxins, SVMPs, SVSPs, and PLA_2_, were found to have edema-forming effects that were optimal at 2 h after envenomation. PLA_2_s had the highest edema-inducing activity, with onset 30 min after envenomation.

**Conclusions:**

Given the importance of SVMPs in altering the integrity of the membrane structure and impairing the blood coagulation system, an antivenom that can specifically neutralize its activity could inhibit the hemorrhage effects of the venoms.

## Introduction

Snakebite envenoming is listed as a priority neglected tropical disease due to the significant burden of mortality and morbidity, which predominantly affects vulnerable populations in the tropical and subtropical regions of the world [1]. Envenomings are one of the leading causes of death and disability in sub-Saharan Africa, Asia, and Latin America [2–4]. Nigeria has the highest burden of snakebite envenomation in sub-Saharan Africa [4]. Recent estimates indicate that approximately 43,000 snakebites occur annually in Nigeria, resulting in around 19,000 deaths [4]. Among the native Nigerian viper species, *Echis ocellatus* and *Bitis arietans* are the most medically important [5, 6]. *E. ocellatus* accounts for approximately 66% of all cases of envenomation [5]. Its range dominates the northern savanna regions and the southern parts of the Oyo and Enugu states [7, 8]. While less frequently found, *B. arietans* also occurs in these same regions [4].

Proteomic and transcriptomic studies have provided information on the composition of viper snake venom. These venoms contain a variety of enzymatic and nonenzymatic protein families, with snake venom metalloproteinases (SVMPs), snake venom serine proteases (SVSPs), and snake venom phospholipase A_2_ (PLA_2_) often predominating [6, 9–12]. Lesser amounts of proteins from other families are also present. Research indicates that these proteins carry out a range of toxic activities in envenomed victims, resulting in local tissue damage and hemorrhage. According to published reports, SVMPs, SVSPs and PLA_2_ have been shown to damage tissues and induce bleeding through their specific enzymatic actions [3, 13–15].

Advancements in toxinology focus on neutralizing venom-induced lethality [3, 16]. Given the diversity of venomous snake species and the pathophysiological characteristics of snake venoms, evaluating the toxic activities of their constituent proteins can help to understand the relative importance of individual components that must be neutralized [3, 17]. This assessment could guide the development of novel and effective treatment strategies based on the 3Rs (Replacement, Reduction, and Refinement) concept, which involves using antitoxin mixtures as replacement or reinforcement for antivenoms [2, 3, 18].

Toxicovenomics incorporates multiomics data into the evaluation of venom toxins [19–21], which may allow the development of improved therapeutic approaches for antivenoms [21–23]. In a recent study, we conducted proteomic analysis of the venoms of Nigerian viper snakes *E. ocellatus* and *B. arietans* [6]. It revealed that metalloproteinases, phospholipases A_2_ and serine proteinases were the primary toxic protein families. The purpose of the current research is to evaluate the hemorrhagic, edematogenic, and in vitro fibrinolytic effects of these main venom protein groups identified in the study. This can help identify protein families that should be prioritized for neutralization by antivenom in snakebite cases, and it can also inform and provide guidance for developing therapeutic inhibitors of snake toxins, paving the way for new treatment options in the future.

## Materials and methods

### Experimental animals

All snake handling and experimental mouse protocols were observed and followed according to the Ahmadu Bello University Committee on Animal Use and Care (approval number: ABUCAUC/2022/048), as well as the revised ARRIVE guidelines. This study used fifty mice weighing 16.2 ± 1.4 g procured from the Faculty of Pharmaceutical Sciences at Ahmadu Bello University in Zaria, Nigeria.

### Snake venoms

This study used venom from adult *E. ocellatus* and *B. arietans* that have been previously reported [6]. Snakes were captured from various regions and housed at the Serpentarium of the Department of Veterinary Pharmacology and Toxicology at Ahmadu Bello University. Venom was extracted manually [24] and those from snakes of the same species were combined. The collected venoms were frozen at −80 °C, lyophilized in a freeze dryer and stored at −20 °C.

### Chemicals and reagents

The following reagents were obtained from Sigma-Aldrich (St. Louis, MI, USA): pure bovine serum albumin (BSA), 1,10-phenanthroline, ethylene diaminetetraacetic acid (EDTA), Bradford reagent, and phenylmethylsulfonyl fluoride (PMSF). Thermo Fisher Scientific (USA) supplied the consumables and chemicals used to prepare the buffers, which were analytical grade.

### Isolation of the venom proteins SVMP, SVSP, and PLA_2_ from crude venom

The crude venoms were fractionated using column chromatography, focusing on the three main venom toxins identified in the proteomic analysis [6]: snake venom metalloproteinase, snake venom serine protease and phospholipase A_2_. Acetone precipitation was performed on each of the crude venoms. Four (4) ml of cold acetone was added to 10 mg/ml of the crude venom. The mixture was incubated at −20 °C for 15 min before being centrifuged at 15,700 × g at 4 °C for 15 min. The protein pellet was air dried and suspended in 100 μl of 1 × PBS buffer at pH 7.4. These served as the protein samples that were fractionated. Two (2) ml of venom at a concentration of 10 mg/ml were loaded onto a DEAE cellulose column (1.5 × 50 cm) preequilibrated with 50 mM phosphate buffer at pH 6. At a flow rate of 0.2 ml/min, the column was eluted stepwise with a NaCl gradient of 0.01–0.1 M. Twenty-five fractions of 2 ml each were collected and tested for metalloproteinase, serine protease, and phospholipase activity. Fractions with similar enzymatic activity were combined and loaded onto a sephadex G-75 column equilibrated with 50 mM phosphate buffer at pH 6.5. The column was eluted using the same buffer at a rate of 1 ml/min. Twenty-five 2 ml fractions were collected and tested for metalloproteinase, serine protease, and phospholipase activity.

### Biochemical assay of venom proteins

The biochemical activities of various venom proteins were analyzed using a Cary 300 UV–visible spectrophotometer (Agilent Technologies, Santa Clara, USA).

### Snake venom metalloproteinase assay

The activity of proteolytic metalloproteinases was measured using a slightly modified method as previously described [25]. Twenty (20) µl of venom protein were mixed with 100 µl of azocasein substrate (10 mg/ml of azocasein dissolved in 25 mM Tris, 150 mM NaCl, 5 mM CaCl_2_, pH 7.4) and incubated at 37 °C for 90 min. The reaction was stopped by adding 200 µl of 5% trichloroacetic acid and centrifuging for 15 min at 3,000 rpm. One hundred (100) µl of supernatant was mixed with 100 µl of 0.5 M NaOH, and casein hydrolysis was measured at 450 nm. The reaction mixture was tested in the presence and absence of 15 mM 1,10 phenanthroline, a metalloproteinase inhibitor. A unit of proteolytic activity was defined as a 0.2 unit change in absorbance per min.

### Snake venom serine protease assay

The substrate solution for serine protease activity was prepared by dissolving Nα-benzoyl-DL-arginine-p-nitroaniline (BApNA, obtained from Sigma-Aldrich) in 5 ml of dimethylsulfoxide (DMSO) and adding 95 ml of 0.05 M Tris–HCl buffer at a pH of 8.2. The assay was carried out with and without the inclusion of 15 mM phenylmethylsulfonyl fluoride. Hydrolysis of BApNA was used to monitor proteolytic activity by mixing 50 μl of the sample with 100 μl of the substrate solution. Activity was determined after 10 min of incubation at 37° C by measuring the release of p-nitroanilide, which absorbs at 405 nm. One unit of protease activity was defined as causing a 0.01/min increase in absorbance [26].

### Snake venom phospholipase A_2_ assay

An enzyme activity assay was performed to measure phospholipase A_2_ (PLA_2_) using an egg yolk substrate solution, as previously described [27]. The egg yolk substrate solution was prepared fresh each time by dissolving egg yolk in a 0.9% saline solution. The assay was carried out at room temperature by incubating 250 μl of the egg yolk substrate solution with 10 μl of PLA_2_. The absorbance was measured at 740 nm after 60 min of incubation. PLA_2_ activity was quantified by determining the amount of PLA_2_ venom required to reduce the turbidity of the solution by 0.01 absorbance units per min at 740 nm, under the conditions tested.

### Protein quantification

The protein concentration of the venoms was quantified using the Bradford method with bovine serum albumin (BSA) serving as standard reference [28]. Replicate aliquots of each sample were incubated for 20 min at 26 °C with 250 μl of Bradford reagent (Sigma-Aldrich, USA) prior to spectrophotometric analysis. Subsequently, absorbance readings at 595 nm were measured on a Cary 300 UV–visible spectrophotometer (Agilent Technologies, Santa Clara, USA) to determine the protein content of venom samples.

### One-dimensional gel electrophoresis

Venom samples were normalized to ensure equivalent protein content prior to separation by 12% sodium dodecyl sulfate–polyacrylamide gel electrophoresis (SDS-PAGE) under non-reducing conditions, as previously described [6]. Aliquots containing 10 µg of protein were loaded into each well and subjected to electrophoresis. After completion, the gels were stained with Coomassie Brilliant Blue G-250 to visualize the protein bands. Gel imaging was performed using the Bio-Rad PharosFX Plus System from the Molecular Imager (California, USA).

### Toxicological analyses

A series of experiments was performed to evaluate the fibrinolytic, edematogenic, and hemorrhagic activities of the venom using mouse models, to gain a clear understanding of the toxicity.

### Determination of hemorrhagic activity

The hemorrhagic activity of crude venom and isolated protein fractions (SVMP, SVSP, and PLA_2_) from *E. ocellatus* and *B. arietans* was determined using the mouse skin test [29]. Adult mice weighing approximately 16.2 ± 1.4 g were divided into nine groups of three mice each. Group 1 received *E. ocellatus* metalloproteinase, Group 2 received *E. ocellatus* serine protease, Group 3 received *E. ocellatus* phospholipase A_2_, Group 4 received *B. arietans* metalloproteinase, Group 5 received *B. arietans* serine protease, Group 6 received *B. arietans* phospholipase A_2_, Group 7 received crude *E. ocellatus* venom, Group 8 received crude *B. arietans* venom, while Group 9 served as controls and was injected with 10 μl of PBS. Each group of mice was injected intradermally into the abdominal region with 10 μg/μl of crude venom or isolated protein. After 2 h, the mice were humanely euthanized using chloroform. The skin was removed and the size of the hemorrhagic lesion on the inner surface was measured horizontally, vertically and diagonally. The average of these measurements was taken as the size of the hemorrhagic lesion. Hemorrhagic activity was expressed as the minimum hemorrhagic dose (MHD), defined as the dose required to induce a hemorrhagic area of 10 mm in diameter. To avoid limitations associated with similar hemorrhagic area sizes exhibiting varying hemorrhage levels [3], the amount of hemoglobin present in each lesion was also quantified [30].

### Determination of venom-induced edema activity

The edema-inducing activities of the venom proteins were evaluated using a modified mouse footpad assay [31]. A separate group of experimental animals, divided into eight groups of three mice each, was subcutaneously injected with 10 μg/μl of venom protein in the right hind footpad and 10 μl of phosphate buffered saline in the left hind footpad as a control. The formation of edema was monitored and measured in various time intervals ranging from 0 min to 5 h after injection of the venom using a digital caliper (Mitutoyo, Japan). The percentage increase in the size of the envenomed foot compared to the size of the phosphate buffered saline-injected foot was used to calculate the formation of edema. A minimum edema-forming dose (MED) was defined as the amount of venom protein required to induce at least 30% edema.

### Determination of fibrinolytic activity

One ml of caprine blood was mixed with 3.8% trisodium citrate and centrifuged at 4,300 rpm for 10 min at 4 °C to obtain platelet-poor plasma (PPP). This test utilized the yellowish supernatant, PPP. Calcium chloride-induced clotting time was used to assess the anticoagulant activity of crude venom and venom proteins in PPP [32]. The control for comparison was a phosphate buffered saline/PPP mixture. One unit of coagulant or anticoagulant activity was defined as an increase or decrease in clotting time of 1 s in PPP incubated with crude venom or venom proteins relative to control PPP.

### Data and statistical analyses

The data were statistically analyzed with a statistical package of social sciences (IBM SPSS Inc. Chicago V 19.0) using one-way analysis of variance with a 95% confidence interval. The results were expressed as mean ± standard error of the mean. A *p*-value of less than 0.05 was used as the threshold for statistical significance. The results of the purification protocol of the enzymes are tabulated as total protein, total activity, specific activity, purification fold, and percentage yield. Graphs of the elution profile were plotted using Sigma Plot software (SPCC Inc., Chicago, IL, USA).

## Results

### One-dimensional SDS-PAGE of the venom of *E. ocellatus* and *B. arietans* proteins

**Fig. 1:**
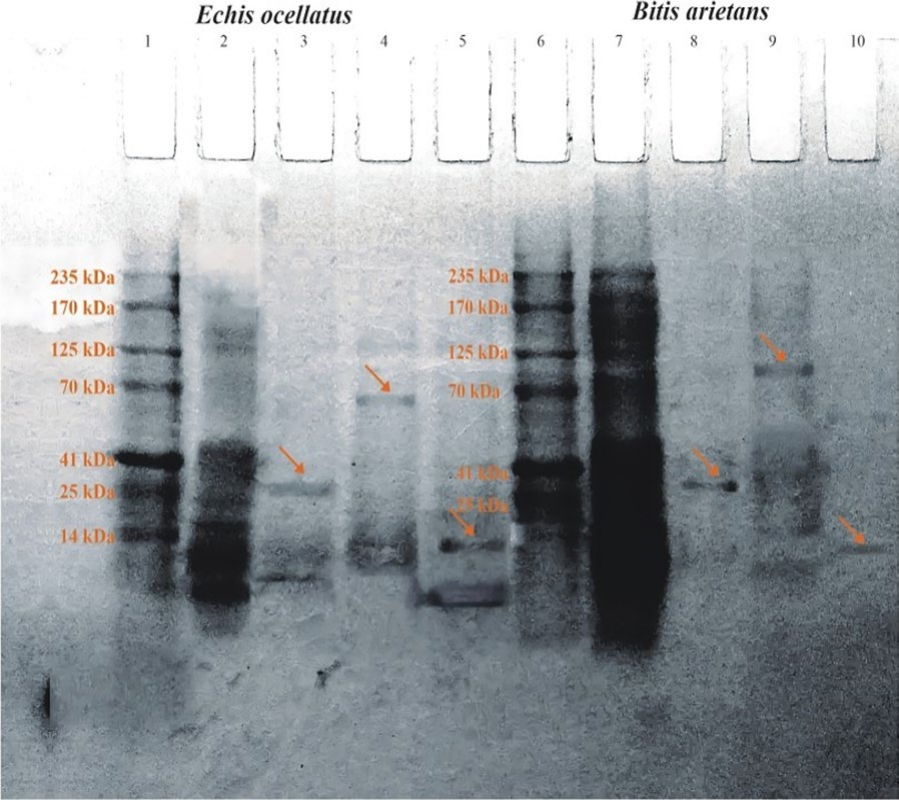
One-dimensional SDS-PAGE of venom proteins of *E. ocellatus* and *B. arietans*

The SDS-PAGE analysis of the venom proteins; snake venom metalloproteinase (SVMP), snake venom serine proteinase (SVSP), and snake venom phospholipase A_2_ (PLA_2_) is shown in Fig. 1. Lanes 1 and 6 contain marker protein standards. Lane 2 contains crude venom from *E. ocellatus*. Lane 3 contains the SVSP fraction with an estimated molecular weight of 28 kDa. Lane 4 contains the SVMP fraction with an estimated molecular weight of 68 kDa. Lane 5 contains the PLA_2_ fraction with an estimated molecular weight of 13 kDa. Lane 7 shows crude venom from *B. arietans*. Lanes 8, 9, and 10 display the SVSP fraction with a molecular weight of 30 kDa, the SVMP fraction with a molecular weight of 95 kDa, and the PLA_2_ fraction with a molecular weight of 13 kDa, respectively.

### Hemorrhagic effects of *E. ocellatus* and *B. arietans* venom and venom proteins

**Fig. 2:**
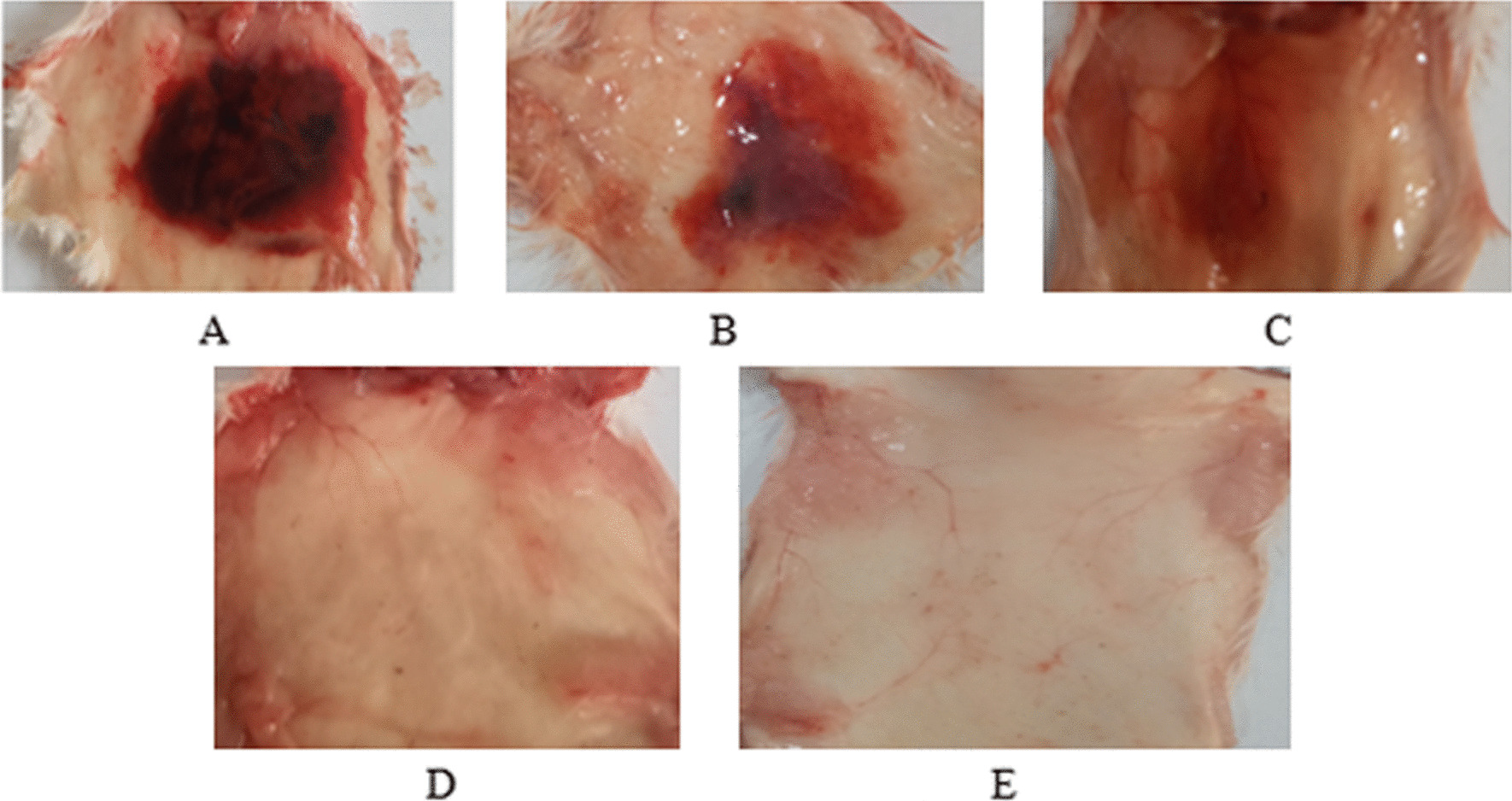
Hemorrhagic lesion/ effects of *E. ocellatus* venom and venom proteins. Hemorrhagic activity was determined 2 h after injecting 10 μg/μl of crude venom or venom proteins (SVMP, SVSP and PLA_2_) intradermally into the abdominal region of mice. Size of hemorrhagic lesion: **A** Crude venom (44 mm), **B** SVMP (26 mm), **C** SVSP (7 mm), **D** PLA_2_ (0 mm), **E** Control (0 mm)

Hemorrhagic lesions caused by crude venom and venom proteins (SVMP, SVSP and PLA_2_) of *E. ocellatus* are shown in Fig. 2. The hemorrhagic lesion measured an average of 26.00 ± 1.00 mm for SVMP, compared to 44.00 ± 1.00 mm for the crude venom. It measured an average of 7.00 ± 1.00 mm for the SVSP. Meanwhile, the PLA_2_ protein did not demonstrate any hemorrhagic effect.

**Fig. 3:**
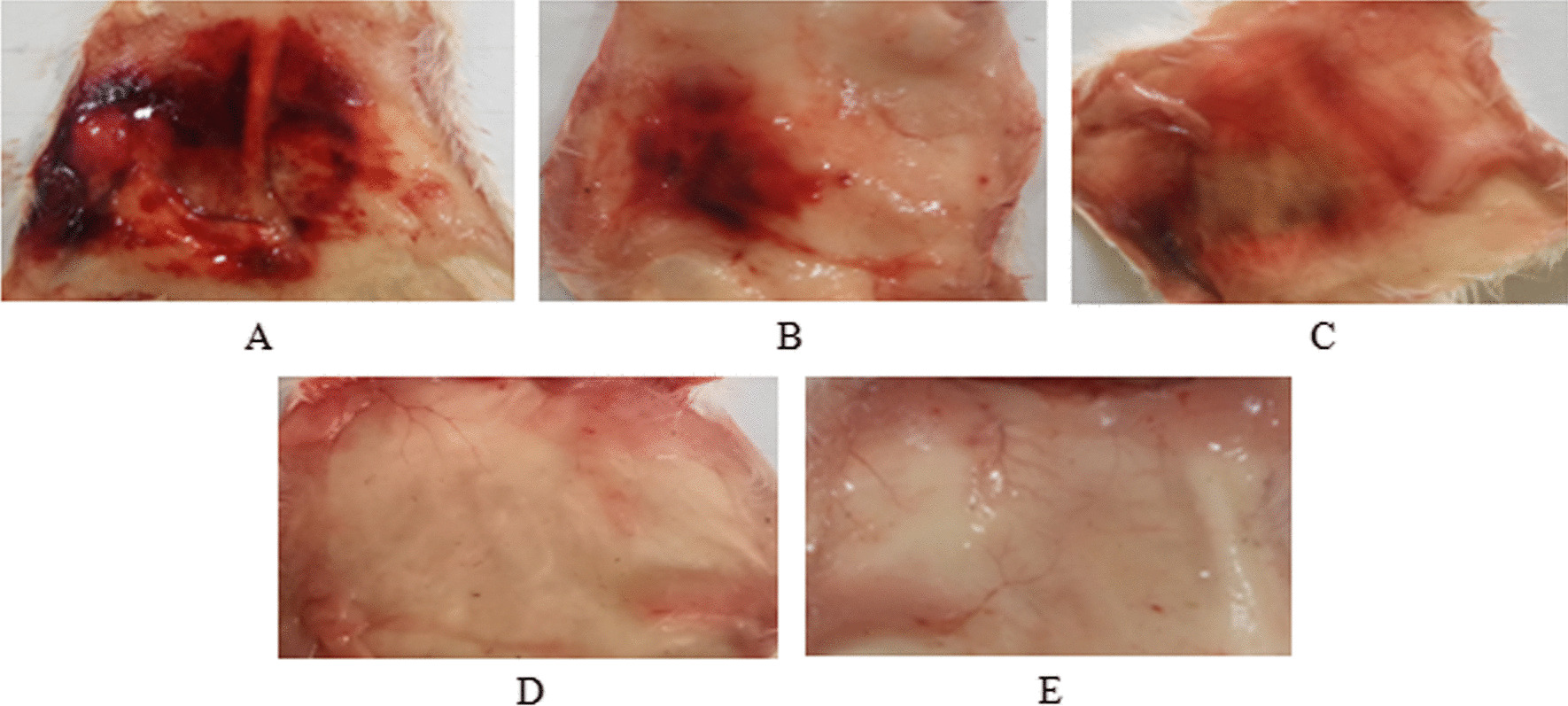
Hemorrhagic lesion/effects of *B. arietans* venom and venom proteins. Hemorrhagic activity was determined 2 h after injecting 10 μg/μl of crude venom or venom proteins (SVMP, SVSP and PLA_2_) intradermally into the abdominal region of mice. Size of hemorrhagic lesion: **A** Crude venom (52 mm), **B** SVMP (21 mm), **C** SVSP (6 mm), **D** PLA_2_ (0 mm), **E** Control (0 mm)

Hemorrhagic lesions caused by *B. arietans* venom and its constituent venom proteins (SVMP, SVSP, and PLA_2_) are shown in Fig. 3. The crude venom produced a hemorrhagic lesion measuring 52.33 ± 1.53 mm. The SVMP fraction resulted in lesions of 21.33 ± 1.52 mm. SVSP caused hemorrhagic lesions averaging 6.33 ± 1.53 mm in size. Meanwhile, PLA_2_ did not appear to induce any hemorrhagic effects according to the data.

### Fibrinolytic effects of *E. ocellatus *and *B. arietans* venoms and venom proteins

The fibrinolytic effects of the venoms and venom proteins of *E. ocellatus* and *B. arietans* are presented in Table 1.

**Table 1:** Fibrinolytic effects of *E. ocellatus* and *B. arietans* venoms and venom proteins

Venom/ venom protein	Plasma recalcification time (min)	
	*Echis ocellatus*	*Bitis arietans*
PPP + PBS + CaCl_2_ (control)	04.10 ± 0.17^a^	03.10 ± 0.10^a^
PPP + CaCl_2_ + PLA_2_	10.00 ± 0.00^b^	12.23 ± 0.15^b^
PPP + CaCl_2_ + SVMP	20.23 ± 0.20^c^	17.20 ± 0.20^c^
PPP + CaCl_2_ + SVSP	30.30 ± 0.26^d^	26.20 ± 0.10^d^
PPP + CaCl_2_ + Crude venom	44.10 ± 0.10^e^	52.20 ± 0.17^e^

Data are mean ± SD from three determinations. Data with different superscripts in a column are significantly different at *p* < 0.05

*PPP* Poor Plasma Protein, *PLA*_*2*_ Snake venom Phospholipase A_2_, *SVMP* Snake venom metalloproteinase, *SVSP* Snake venom serine protease

### Edematogenic activity of the *E. ocellatus* and *B. arietans* venoms and venom proteins

The edematogenic effects of crude venom and venom proteins of *E. ocellatus* are presented in Table 1. The venom demonstrated time-dependent properties for inducing edema. Both the crude venom and SVSP fractions caused edema 30 min after envenomation. Meanwhile, the SVMP and PLA_2_ fractions induced edema 60 min later. In particular, the PLA_2_ fraction recorded the highest edema value of 5.84 ± 0.01 mm at 120 min.

According to the results of the study, the crude *B. arietans* venom caused edema within 1 h of envenomation. The SVMP and SVSP fractions induced edema within 120 min. The onset of the edema of the PLA_2_ fraction ranged from 30 min to 3 h, with a peak at 2 h. Mortality rates for the crude venom group were recorded between 3 and 5 h after envenomation.

### Elution profile of *Echis ocellatus* venom proteins from diethylaminoethyl cellulose

**Fig. 4:**
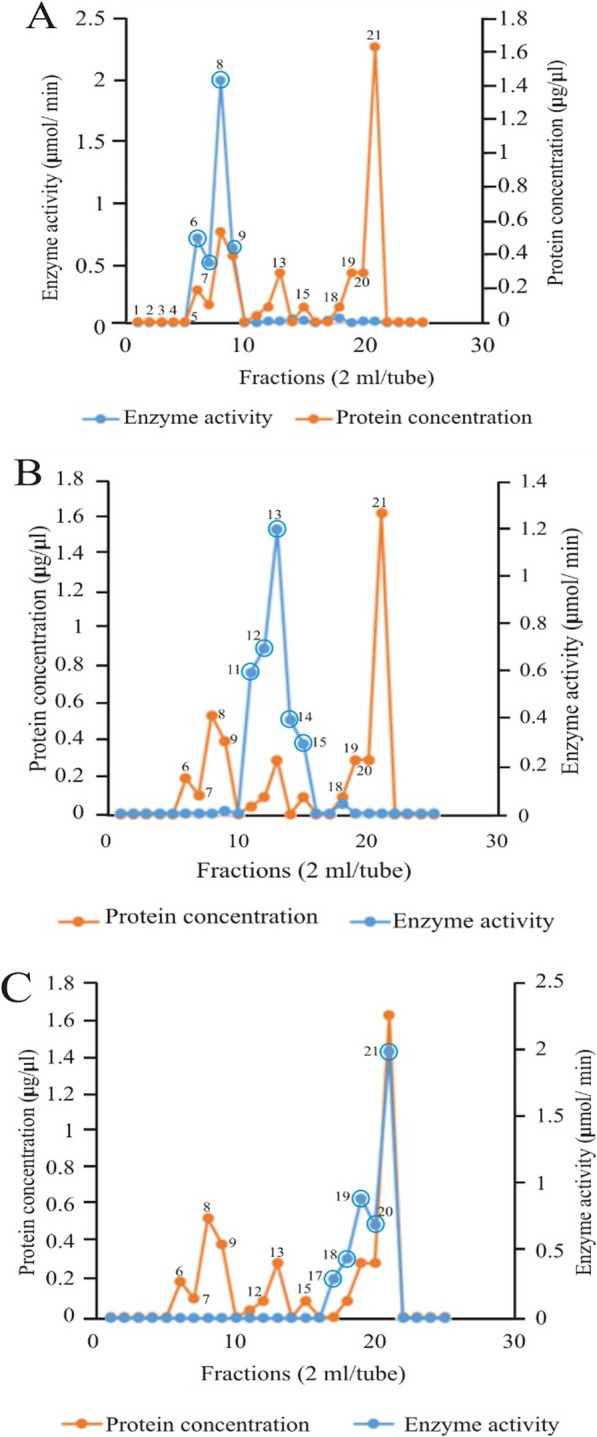
Elution profile of *Echis ocellatus* venom proteins from DEAE cellulose. **A** Snake venom metalloproteinase (SVMP) fractions (Peaks 6, 7, 8, and 9), **B** Snake venom serine protease (SVSP) fractions (Peaks 11, 12, 13, 14 and 15), **C** Snake venom phospholipase A_2_ (PLA_2_) fractions (Peaks 17, 18,19, 20 and 21)

Figure 4 shows the elution profile of *E. ocellatus* venom from diethylaminoethylcellulose (DEAE). 0.01–0.1 M sodium chloride gradients were used to elute the column. The peaks show that snake venom metalloproteinase (SVMP) was eluted in fractions 6, 7, 8, and 9 (panel A). Snake venom serine protease (SVSP) was eluted in fractions 11, 12, 13, 14, and 15 (panel B), while phospholipase A_2_ (PLA_2_) was eluted in fractions 17, 18, 19, 20, and 21 (panel C). proteins other than the protein of interest appeared to be present in the peaks of 1–4 and 13–21 (panel A), 6–9, 18–21 (panel B) and 6–15 (panel C).

### Elution profile of *Echis ocellatus* snake venom metalloproteinase (SVMP) from Sephadex G-75 column

**Fig. 5:**
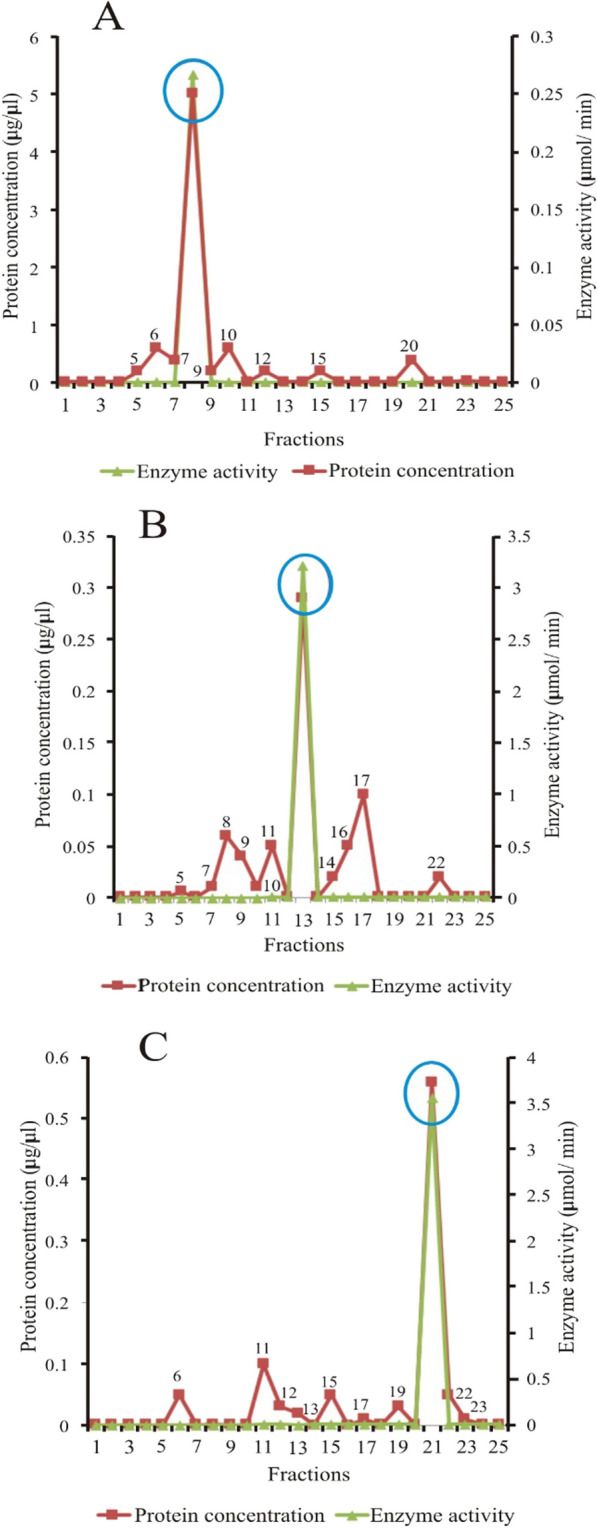
Elution profile of *Echis ocellatus* venom proteins from Sephadex G-75 columns. **A** Snake venom metalloproteinase (SVMP), **B** Snake venom serine protease (SVSP), **C** Snake venom phospholipase A_2_ (PLA_2_)

The 6, 7, 8, and 9 fractions of the DEAE column with metalloproteinase activity were pooled, loaded on a Sephadex G-75 column, and eluted with phosphate buffered saline (PBS, pH 7.4). As shown in Fig. 5A, SVMP was eluted in fraction 8. Other proteins that were eluted alongside SVMP are represented by the eight minor peaks (peaks 5–7, and 9–20) shown in the figure. However, they did not exhibit metalloproteinase activity.

### Elution profile of the snake venom serine protein (SVSP) of *Echis ocellatus* from Sephadex G-75 column

Similarly, fractions 11, 12, 13, 14, and 15 from the DEAE column were pooled and loaded on a Sephadex G-75 column, which was then eluted with PBS (pH 7.4). As illustrated in Fig. 5B, SVSP was eluted in fraction 13. There were 9 peaks of interfering proteins (peaks 5, 7–11, 15–17, and 22), without protease activity.

### Elution profile of ***Echis ocellatus*** venom phospholipase A_2_ (PLA_2_) from Sephadex G-75 column

Likewise, fractions with phospholipase activity from the DEAE column (17, 18, 19, 20, and 21) were pooled and loaded onto a Sephadex G-75 column before being eluted with PBS (pH 7.4). PLA_2_ from the venom of *E. ocellatus* was eluted in fraction 21, as shown in Fig. 5C. The minor proteins eluted as shown (peaks 6, 11, 13, 15, 17, 19, 22, and 23) lacked phospholipase activity and were thus considered as interfering proteins.

### Elution profile of *Bitis arietans* venom proteins from diethylaminoethylcellulose

**Fig. 6:**
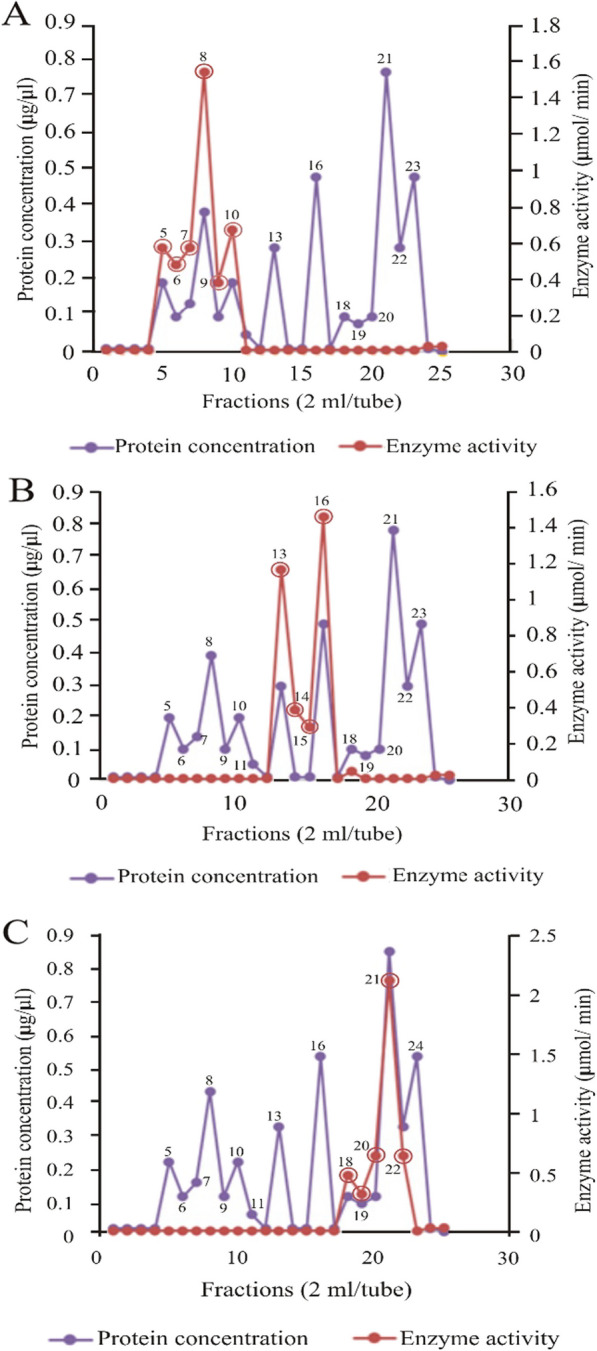
Elution profile of *Bitis arietans* venom proteins from DEAE cellulose. **A** Snake venom metalloproteinase (SVMP) fractions (5, 6, 7, 8, 9 and 10), **B** Snake venom serine protease (SVSP) fractions (Peaks 13, 14, 15 and 16), **C** Snake venom phospholipase A_2_ (PLA_2_) fractions (Peaks 18, 19, 20, 21 and 22)

The elution profile of *B. arietans* venom proteins from a diethylaminoethyl (DEAE) cellulose column is shown in Fig. 6. Sodium chloride gradients of 0.01–0.1 M were used to elute the column. *B. arietans* SVMP was eluted in fractions 5, 6, 7, 8, 9, and 10 of the 25 fractions collected (panel A), as shown by the peaks. *B. arietans* SVSP was eluted in fractions 13, 14, 15, and 16 (panel B), while Phospholipase A_2_ was eluted in fractions 18, 19, 20, 21, and 22 (panel C). Fractions with the same activity were pooled and subjected to a second purification step using Sephadex G-75. Other proteins were found in the fractions in addition to the protein of interest, as indicated by peaks 13–23 (panel A), 5–11, 18–23 (panel B), 5–16, and 24 (panel C).

### Elution profile of *Bitis arietans* venom metalloproteinase from Sephadex G-75 column

**Fig. 7:**
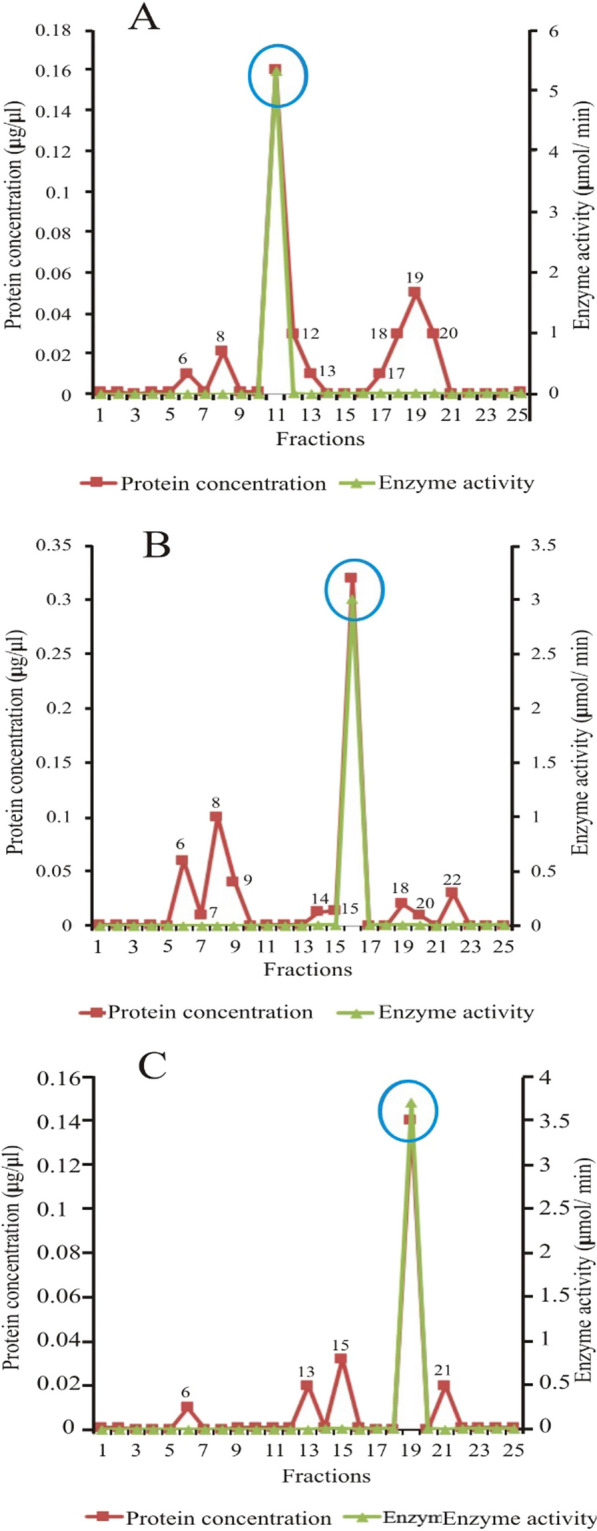
Elution profile of *Bitis arietans* venom proteins from Sephadex G-75 columns. **A** Snake venom metalloproteinase (SVMP), **B** Snake venom serine protease (SVSP), **C**: Snake venom phospholipase A_2_ (PLA_2_)

The elution profile of *B. arietans* venom metalloproteinase (SVMP) from a Sephadex G-75 column is shown in Fig. 7A. PBS (pH 7.4) was used to elute the column, and SVMP was eluted in fraction 11. Other proteins (peaks 6, 8, 12, 13, 17–20) were present, but they lacked metalloproteinase activity.

### Elution profile of *Bitis arietans* venom serum protein (SVSP) from Sephadex G-75 column

As shown in Fig. 7B, *B. arietans* venom SVSP was eluted from the Sephadex G-75 column in fraction 16. Peaks 6–9, 14, 15, 19, 20, and 22 were other proteins eluted but without protease activity.

### Elution profile of ***Bitis arietans*** venom phospholipase A_2_ (PLA_2_) from Sephadex G-75 column

Figure 7C shows the elution profile of PLA_2_ of the venom of *B. arietans.* As shown in the figure, it was eluted with PBS (pH 7.4) in fraction 19. Other proteins that eluted as shown (peaks 6, 13, 15, and 21) lacked phospholipase activity.

### Purification profiles of metalloproteinase (SVMP), serine protease (SVSP) and phospholipase A_2_ (PLA_2_)

The *E. ocellatus* venom proteins were purified in three steps: acetone precipitation, ion exchange chromatography, and gel filtration. The purification profiles for SVMP, SVSP, and PLA_2_ are shown in Table 4. The total protein concentration of SVMP was 0.32 µg, the percentage yield was 25.63, the purification fold was 1.89, and the specific activity was 5.13 µmol/min/mg. SVSP was purified to a purification fold of 1.46 and a total protein concentration of 0.39 µg. The protein had a specific activity of 3.26 µmol/min/mg, a percentage yield of 23.43, and a total activity of 1.27 µmol/min. PLA_2_ had a total protein concentration of 0.56 µg, a yield of 42.07%, a specific activity of 3.55 µmol/min/mg, and was purified to a purification fold of 1.78.

Similarly, the purification profiles of *B. arietans* SVMP, SVSP, and PLA_2_ are shown in Table 5. SVMP had 0.16 µg of total protein, 0.85 µmol/min of total activity, and 5.31 µmol/min/mg of specific activity after gel filtration. The protein was purified to a purification fold of 2.55 and had a yield of 15.75%. SVSP was purified to 1.68 purification fold with a yield of 2.30%. It had 0.32 µg total protein, 0.96 µmol/min total activity and 3.00 µmol/min/mg specific activity. PLA_2_ contained 0.14 µg of total protein and was purified to a purification fold of 3.35 with a yield of 17.78%. The protein had a total activity of 0.53 µmol/min and a specific activity of 3.79 µmol/min/mg. The enzyme purification profile was formulated using the process outlined in Eq. 1.

$$\begin{gathered} {\text{Total}}\,{\text{Protein}}\, = \,\frac{{{\text{Total}}\,{\text{Activity}}}}{{{\text{Specific}}\,{\text{Activity}}}} \hfill \\ {\text{Total}}\,{\text{Activity}}\, = \,{\text{Specific}}\,{\text{Activity}}\, \times \,{\text{Total}}\,{\text{Protein}} \hfill \\ {\text{Specific}}\,{\text{Activity}}\, = \,\frac{{{\text{Enzyme}}\,{\text{Activity}}\,\left( {{\text{Total}}\,{\text{Activity}}} \right)}}{{{\text{Total}}\,{\text{Protein}}}} \hfill \\ {\text{Purification}}\,{\text{Fold}}\, = \,\frac{{{\text{Specific}}\,{\text{Activity}}\,{\text{of}}\,{\text{Protein}}\,{\text{at}}\,{\text{a}}\,{\text{Purification}}\,{\text{Step}}}}{{{\text{Original}}\,{\text{Specific}}\,{\text{Activity}}\,\left( {{\text{Crude}}} \right)}} \hfill \\ {\text{Percentage}}\,{\text{Yield}}\, = \,\frac{{{\text{Total}}\,{\text{Activity}}\,{\text{of}}\,{\text{protein}}\,{\text{at}}\,{\text{a}}\,{\text{step}}}}{{{\text{Total}}\,{\text{Activity}}\,{\text{of}}\,{\text{Original}}\,{\text{Purification}}\,\left( {{\text{Crude}}} \right)}}\, \times \,100 \hfill \\ \end{gathered}$$

Protein purification table calculations formular: Eq. 1.

## Discussion

This study describes the main toxins responsible for the predominant toxicological effects in the venoms of the Nigerian saw-scaled viper *Echis ocellatus* and puff adder *Bitis arietans*. Previous proteomic analyzes revealed that metalloproteinases, phospholipase A_2_s, and serine proteases were the main toxins in these vipers [6], which is consistent with viper venoms from other regions [3, 33, 34]. Since venom proteins can act synergistically or independently to cause tissue damage [3, 35], this study performed toxicological studies on snake venom metalloproteinases (SVMPs), snake venom serine proteases (SVSPs), and snake venom phospholipases A_2_ (PLA_2_) to determine their relative importance for toxicity. The toxicological activity of these venom components determined by the mouse skin test (Figs. 2B and 3B) revealed that SVMP is primarily a hemorrhagic protein due to the damage to the capillary vessels that it causes [36, 37]. SVMPs are known to cause hemorrhage by cleaving the basement membrane of capillary vessels, causing the vessels to weaken. This results in the detachment of endothelial cells from the basement membrane, thinning it and eventually disrupting the capillary walls, leading to extravasation of blood [37, 38].

When SVMPs degrade the integrity of cell membranes, the tissues are more susceptible to infiltration by other venom toxins. It leads to pathophysiological conditions caused by a complex mixture of toxins in the venom [3, 39]. This underscores the pathological importance of neutralizing SVMPs as one of the key components in the event of envenomation. The hemorrhagic lesions in Figs. 2B and 3B demonstrate that venoms have a high propensity for hemorrhage, attributable to SVMP. This aligns with proteomic data [6], especially in the venom of *E. ocellatus,* where SVMPs represented the highest percentage (34.84%) of total venom content. Therefore, the hemorrhagic effects of *E. ocellatus* SVMP observed in this study indicate that the envenomation of *E. ocellatus* would likely prove more lethal than the envenomation of *B. arietans.* Consequently, any antivenom capable of neutralizing SVMPs has the potential to significantly mitigate the toxic effects of venom. In addition to inducing hemorrhage, it also interferes with blood coagulation pathways, and *E. ocellatus* inhibits coagulation to a greater extent than *B. arietans*. The results of this study corroborate previous reports that SVMPs affect hemostasis by altering coagulation, promoting hemorrhage [40, 41] by modulating key enzymes such as fibrinogenase and fibrolase [42, 43]. Evaluation of edema revealed involvement of SVMP in inflammatory processes for both venoms. However, the onset of edema was more rapid for *E. ocellatus* (Table 2) than for *B. arietans* (Table 3). This is consistent with previous findings of SVMP inflammatory properties [41, 44, 45].

**Table 2:** Edematogenic activity of *E. ocellatus* crude venom and venom proteins

Time (Min)	Crude venom		SVMP fraction		SVSP fraction		PLA_2_ fraction	
	Envenomed foot (mm)	Control	Envenomed foot (mm)	Control	Envenomed foot (mm)	Control	Envenomed foot (mm)	Control
0	1.17 ± 0.01^a^	1.11 ± 0.00^a^	1.58 ± 0.01^a^	1.57 ± 0.00^a^	1.44 ± 0.00^a^	1.41 ± 0.00^a^	1.73 ± 0.01^a^	1.60 ± 0.00^a^
30	4.65 ± 0.01^g^	1.11 ± 0.01^a^	3.17 ± 0.01^c^	1.57 ± 0.01^a^	4.25 ± 0.01^g^	1.42 ± 0.00^a^	3.82 ± 0.01^d^	1.61 ± 0.01^a^
60	3.34 ± 0.01^e^	1.11 ± 0.00^a^	4.03 ± 0.01^f^	1.57 ± 0.01^a^	3.77 ± 0.01^f^	1.42 ± 0.00^a^	4.34 ± 0.00^e^	1.60 ± 0.01^a^
120	3.44 ± 0.01^f^	1.12 ± 0.01^a^	4.03 ± 0.00^f^	1.57 ± 0.00^a^	3.68 ± 0.00^e^	1.42 ± 0.00^a^	5.84 ± 0.01^f^	1.61 ± 0.01^a^
180	3.17 ± 0.01^c^	1.11 ± 0.00^a^	3.30 ± 0.00^e^	1.57 ± 0.00^a^	3.51 ± 0.01^d^	1.42 ± 0.00^a^	3.82 ± 0.01^d^	1.61 ± 0.01^a^
240	3.21 ± 0.01^d^	1.12 ± 0.01^a^	2.84 ± 0.01^b^	1.57 ± 0.00^a^	2.54 ± 0.02^b^	1.42 ± 0.00^a^	3.31 ± 0.01^b^	1.61 ± 0.00^a^
300	2.68 ± 0.00^b^	1.11 ± 0.00^a^	3.24 ± 0.00^d^	1.57 ± 0.00^a^	3.35 ± 0.01^c^	1.41 ± 0.00^a^	3.62 ± 0.01^c^	1.62 ± 0.01^a^

Data are mean ± SD from three determinations. Data with different superscripts in a column are significantly different at *p* < 0.05

**Table 3:** Edematogenic activity of crude venom and venom proteins of *B. arietans*

Time (Min)	Crude venom		SVMP fraction		SVSP fraction		PLA_2_ fraction	
	Envenomed foot (mm)	Control	Envenomed foot (mm)	Control	Envenomed foot (mm)	Control	Envenomed foot (mm)	Control
0	1.12 ± 0.00^a^	1.13 ± 0.00^a^	1.81 ± 0.01^a^	1.72 ± 0.01^a^	2.11 ± 0.01^a^	2.10 ± 0.01^a^	1.70 ± 0.00^a^	1.71 ± 0.01^a^
30	3.24 ± 0.01^b^	1.12 ± 0.00^a^	3.51 ± 0.00^d^	1.72 ± 0.00^a^	3.60 ± 0.00^c^	2.09 ± 0.02^a^	4.04 ± 0.00^d^	1.72 ± 0.01^a^
60	4.42 ± 0.01^d^	1.13 ± 0.00^a^	3.93 ± 0.02^f^	1.73 ± 0.01^a^	3.74 ± 0.00^d^	2.10 ± 0.01^a^	4.65 ± 0.00^e^	1.72 ± 0.02^a^
120	3.61 ± 0.00^c^	1.13 ± 0.00^a^	4.57 ± 0.00^g^	1.73 ± 0.02^a^	5.47 ± 0.00^g^	2.08 ± 0.01^a^	5.60 ± 0.00^g^	1.72 ± 0.01^a^
180	×	×	3.72 ± 0.00^e^	1.73 ± 0.00^a^	3.85 ± 0.00^e^	2.07 ± 0.02^a^	4.84 ± 0.00^f^	1.71 ± 0.00^a^
240	×	×	2.48 ± 0.00^b^	1.73 ± 0.01^a^	2.82 ± 0.00^b^	2.08 ± 0.00^a^	3.34 ± 0.00^c^	1.73 ± 0.01^a^
300	×	×	2.52 ± 0.00^c^	1.73 ± 0.01^a^	4.13 ± 0.00^f^	2.07 ± 0.01^a^	3.11 ± 0.00^b^	1.71 ± 0.00^a^

Data are mean ± SD from three determinations. Data with different superscripts in a column are significantly different at *p* < 0.05. × indicates mortality

A study examining the functional analysis of Brazilian *Bothrops atrox* venom proteins found that metalloproteinase produced a hemorrhagic effect with a diameter of 16 mm at a concentration of 10 µg [46]. For comparison, the venom of the Nigerian snake *E. ocellatus* caused a larger hemorrhagic diameter of 26 mm also at 10 µg, while the venom of *B. arietans* resulted in a 21 mm. These divergent results can be attributed to several potential factors. Differences in venom protein expression levels between snake populations that inhabit disparate regions worldwide could explain the observed variances in hemorrhagic effect. Local evolutionary pressures and environmental conditions may have shaped adaptive changes in venom composition and toxicity over time between snake species in different geographic locales.

The primary toxicity mechanism of SVSPs in both venoms involves anticoagulation. They exhibited longer plasma recalcification times than crude venoms (Table 1), suggesting that SVSPs are hemotoxic proteins. This effect is more pronounced in *E. ocellatus*, which has a longer clotting time than in *B. arietans*. SVSPs have exact toxicity by acting as thrombin-like enzymes or plasminogen activators, which eliminate fibrin in blood clots and contribute significantly to coagulopathy [14, 47]. Activation, inactivation, or depletion of coagulation factors prevents blood clotting, resulting in noncoagulable blood and bleeding [47]. In addition to altering the hemostatic system as their primary toxicity mechanism, SVSPs also cause edema in *E. ocellatus* (Table 2) and *B. arietans* (Table 3). This is consistent with reports in the literature of SVSP inducing edema, although the precise mechanism remains unknown [48].

According to mouse footpad analyses, PLA_2_ functions primarily as a protein that induces edema (Tables 2 and 3). It causes edema by inducing thermal allodynia and mechanical hyperalgesia [49, 50]. In addition to its ability to induce edema, it also possesses anticoagulant properties, with coagulation times of 10 and 12 min observed in the venom of *E. ocellatus* and *B. arietans,* respectively (Tables 2 and 3). These anticoagulant effects provide validity to reports that PLA_2_ increases hemorrhage by inhibiting the blood coagulation process [1, 49]. The results of the elution and purification processes of the venom proteins are presented in Figs. 4, 5, 6, 7. Subsequently, Tables 4 and 5 show the results of the purification of the enzymes as the specific activity, total activity, yield, and purification factor. Ideally, protocols to purify enzymes from snake venom should achieve several key objectives, such as obtaining enzymes at a high level of purity. This would enhance both the specific activity and purification fold. The specific activities of the enzymes of both venoms were not efficiently enhanced as a result of the protocol used for enzyme purification. As such, one of the limitations of this study was the purification protocol.

**Table 4:** Purification profile of *E. ocellatus* metalloproteinase, serine protease and phospholipase A_2_ fractions

Venom fraction	Purification step	Total protein (µg)	Total activity (µmol/min)	Specific activity (µmol/min/mg)	Purification fold	Yield (%)
SVMP						
	Crude venom	2.36	6.40	2.71	1.00	100.00
	Acetone precipitation	0.92	2.64	2.87	1.06	41.30
	Ion exchange chromatography on DEAE cellulose	0.54	1.99	3.69	1.36	31.10
	Gel filtration on Sephadex G-75	0.32	1.64	5.13	1.89	25.63
SVSP						
	Crude venom	2.36	5.42	2.22	1.00	100.00
	Acetone precipitation	0.95	2.64	2.77	1.24	48.70
	Ion exchange chromatography on DEAE cellulose	0.50	1.50	3.00	1.35	27.70
	Gel filtration on Sephadex G-75	0.39	1.27	3.26	1.46	23.43
PLA_2_						
	Crude venom	2.36	4.73	2.00	1.00	100.00
	Acetone precipitation	1.82	3.94	2.16	1.10	83.30
	Ion exchange chromatography on DEAE cellulose	1.43	3.62	2.53	1.27	76.56
	Gel filtration on Sephadex G-75	0.56	1.99	3.55	1.78	42.07

**Table 5:** Purification profile of *Bitis arietans* metalloproteinase, serine protease and phospholipase A_2_ fractions

Venom fraction	Purification step	Total protein (µg)	Total activity (µmol/min)	Specific activity (µmol/min/mg)	Purification fold	Yield (%)
SVMP						
	Crude venom	2.64	5.40	2.08	1.00	100.00
	Acetone precipitation	0.83	2.41	2.90	1.39	48.20
	Ion exchange chromatography on DEAE cellulose	0.40	1.62	4.05	1.95	30.00
	Gel filtration on Sephadex G-75	0.16	0.85	5.31	2.55	15.75
SVSP						
	Crude venom	2.64	4.73	1.79	1.00	100.00
	Acetone precipitation	0.93	2.54	2.73	1.52	53.69
	Ion exchange chromatography on DEAE cellulose	0.52	1.50	2.88	1.58	31.70
	Gel filtration on Sephadex G-75	0.32	0.96	3.00	1.68	20.30
PLA_2_						
	Crude venom	2.64	2.98	1.13	1.00	100.00
	Acetone precipitation	1.62	2.24	1.38	1.22	77.24
	Ion exchange chromatography on DEAE cellulose	1.09	1.80	1.65	1.46	60.40
	Gel filtration on Sephadex G-75	0.14	0.53	3.79	3.35	17.78

## Conclusions

Identification and understanding of common venom toxins and their toxicological effects can be critical in avoiding treatment failures and developing new therapeutic approaches for antivenom design [3, 21, 23, 51]. When comparing the hemorrhagic effects of the SVMP fraction with crude venom, the fraction generated hemorrhagic lesions of 26.00 ± 1.00 mm in *E. ocellatus* and 21.33 ± 1.52 mm in *B. arietans*. Considering the importance of SVMP in toxicity by disrupting membrane integrity, we conclude that SVMPs represent a high-priority target for neutralization. Therefore, the development of therapeutics aimed at SVMP inhibition may serve to limit tissue damage after envenomation by blocking this initial breach of the membrane barrier. 

## Data Availability

The data sets used and/or analyzed during the current study are available from the corresponding author on reasonable request.
